# Two sexes respond equally to food restriction in a sexually dimorphic but not body mass dimorphic jumping spider

**DOI:** 10.1002/ece3.8112

**Published:** 2021-09-21

**Authors:** Qin Li, Jing‐Xin Liu, Bing Dong, Rong Xiao, Zhanqi Chen

**Affiliations:** ^1^ Guizhou Provincial Key Laboratory for Agricultural Pest Management of the Mountainous Region Institute of Entomology Guizhou University Guiyang China; ^2^ CAS Key Laboratory of Tropical Forest Ecology Xishuangbanna Tropical Botanical Garden Chinese Academy of Sciences Mengla China; ^3^ Environmental Education Center Xishuangbanna Tropical Botanical Garden Chinese Academy of Sciences Mengla China

**Keywords:** fecundity selection, life history traits, sex role, sex‐specific response, sexual difference

## Abstract

Natural selection favors animals that evolve developmental and behavioral responses that buffer the negative effects of food restrictions. These buffering responses vary both between species and within species. Many studies have shown sex‐specific responses to environmental changes, usually in species with sexual size dimorphism (SSD), less found in species with weak or no SSD, which suggests that sizes of different sexes are experiencing different selections. However, previous studies usually investigated development and behavior separately, and the balanced situation where males and females of sexually dimorphic species respond in the same way to food restriction remains little known. Here, we investigated this in *Phintelloides versicolor* (Salticidae) that presents sexual dimorphism in color and shape but weak SSD. We examined whether food restriction induced the same responses in males and females in development duration, adult body size and weight, daily time allocated to foraging, and hunting. We found food restriction induced similar responses in both sexes: both exhibited longer development duration, smaller adult body size and weight, higher probability of staying outside nests and noticing prey immediately, and higher hunting success. However, there were sexual differences regardless of food condition: females showed faster development, smaller adult body size, higher probability of staying outside of nests, and higher hunting success. These indicated the differential selection on male and female sizes of *P. versicolor* could be under a balanced situation, where males and females show equal developmental and behavioral plasticity to environmental constraints.

## Graphical abstract

Two sexes respond equally to food restriction in a sexually dimorphic but not the body mass dimorphic jumping spider.

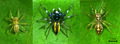

## 1 INTRODUCTION

Food resources are indispensable to survival and reproduction for animals. Food restriction has effects on a series of life history traits, including development duration (Berrigan & Charnov, 1994; George et al., 2002; Nylin & Gotthard, 1998; Uhl et al., 2004), daily time allocated to foraging (Lenski, 1984) and hunting behavior (Aguilar‐Argüello & García‐Chávez, 2015). However, in the wild, animals usually experience a lack of food resources, which favors the evolution of adaptive strategies that mitigate the effects of food or nutrition restriction (Metcalfe & Monaghan, 2001; Nylin & Gotthard, 1998). These adaptive strategies not only differ among species, but also within species (Cordellier et al., 2020; Leimar et al., 1994; Neumann et al., 2017; Nylin & Gotthard, 1998; Uhl et al., 2004). Understanding the intraspecific differences in adaptive strategies under food restriction might help us understand how selection shapes the intersexual or inter‐age differences.

Under food restriction, natural selection can favor developmental and behavioral strategies to compensate the negative effects of starving. The trade‐off between developmental time and body size can favor a relatively large size by delaying sexual maturation (Quiñones‐Lebrón et al., 2021; Uhl et al., 2004; Vertainen et al., 2000). For behavioral strategies, time allocated to foraging can be increased (Abrams, 1991; Lenski, 1984; Weterings et al., 2018), and individuals might also adjust their foraging ways to enhance the success rates (Aguilar‐Argüello & García‐Chávez, 2015). Either developmental or behavioral strategies under food restriction were reported before. However, few studies combined them (Uhl et al., 2004), which is needed for a full understanding of the evolution of the life history strategies in response to food restriction considering the interactions between these strategies.

The importance of food resources may differ between sexes and/or age groups (Cordellier et al., 2020; Nylin & Gotthard, 1998; Uhl et al., 2004). Because of the sex roles (Fairbairn, 1997), even under same food stress, in species with maternal care, males usually would take strategies to maximize mating success, while females usually would rather ensure both the reproduction and parenting success (Trivers, 1972). This different importance of food may also exhibit between juveniles and adults, as adults need food for maintenance and reproductive activities, while for juveniles the urgent need for food is mostly to canalize resources to their development. Thus, there could be both sex‐specific and age‐specific strategies under food restriction for the same species. Sex‐specific plasticity to environmental variation including food availability were assumed to be a cause of intraspecific variation of sexual size dimorphism (SSD) (Fernández‐Montraveta & Moya‐Laraño, 2007), and, usually, the individuals of the sex suffering higher cost of departing from the optimal body size is expected to show higher canalization in body size (Fernández‐Montraveta & Moya‐Laraño, 2007). The type and magnitude of selection on body size can differ between males and females (Castillo & Núñez‐Farfán, 2008; Shine, 1989). Male body size is mainly under sexual selection, and female under fecundity selection (Blanckenhorn, 2005). Many studies found male body size varies more intensively across populations or environmental conditions than female's (Fischer & Fiedler, 2000; Morin et al., 1999; Nylin & Gotthard, 1998; Stillwell & Fox, 2007) and owed the reasons to a stronger stabilizing selection on female body size as it is closely related to fecundity (“fecundity selection hypothesis”) (Uhl et al., 2004). However, there was also evidence showing the opposite, in which male body size is more canalized (Leimar et al., 1994; Nylin & Gotthard, 1998; Turnbull, 1965) and the main driving force might be a stronger selection on male body size; thus the “sexual selection hypothesis” has been proposed (Teder & Tammaru, 2005). Furthermore, even species without SSD in normal condition could exhibit SSD when the condition changes, such as male butterflies (*Lycaena hippothoe*) matured earlier with smaller body size when temperature increased while females kept a size not different from when the temperature did not change (Fischer & Fiedler, 2000). The fact that males and females in many species showed different responses to environmental variation (Stillwell et al., 2009) even in species with no SSD (Fischer & Fiedler, 2000) indicates that whether sex‐specific plasticity exist or not may depend on the consequences of different selective forces on males and females. The sex with either larger or smaller or same body size can exhibit a higher degree of developmental plasticity (Teder & Tammaru, 2005), or different sexes of species with limited size difference could show same degree of plasticity in which males and females respond to environmental changes equally so that intraspecific SSD can remain same across conditions (Oudin et al., 2015).

Female‐biased SSD is the common case in arthropods (Blanckenhorn et al., 2007; Hochkirch et al., 2008), and studies demonstrating sex‐specific responses to food restriction mostly used species with moderate to high SSD (Cordellier et al., 2020; Fernández‐Montraveta & Moya‐Laraño, 2007; Livingston et al., 2014; Neumann et al., 2017), in which sex with smaller body size usually would respond more strongly, hence mostly either “sexual selection hypothesis” or “fecundity selection hypothesis” would be the explanation (Blanckenhorn, 2005). There are a few studies that used species with weak or no SSD to investigate sex‐specific responses to food restriction (Leimar et al., 1994; Rohner et al., 2018; Uhl et al., 2004; Vertainen et al., 2000). For species with weak or no SSD, the body size could be a result of either equal degree of condition dependence of both sexes in whatever condition or sexually equal condition dependence only in good condition (where SSD would become significant in low condition) (Bonduriansky, 2007a, 2007b), or it can be the result of similar degree of canalization in both sexes. Thus, species with weak or even no SSD but showing both signs of sexual selection and fecundity selection would offer a balanced case to analyze the differences between males and females in life history traits, as well as the responses of these traits to environmental changes.

Jumping spiders (Salticidae) would offer many species appropriate for this analysis. Although jumping spiders have one of the lowest sexual size dimorphism (SSD) among all the spider families (Vollrath & Parker, 1992), there is substantial evidence of sexual selection on male traits related to courtship or male–male competition, including color (Li et al., 2008), appendage size (Tedore & Johnsen, 2012) and courtship movement (Jackson, 1977). Evidence suggests that female jumping spiders are selected by fecundity (Kuntner & Coddington, 2020; Uhl et al., 2004). Thus, we selected the jumping spider *Phintelloides versicolor* (Kanesharatnam & Benjamin, 2019; Koch, 1846) with weak SSD but remarkable sexual color dimorphism to investigate (1) whether males and females would respond in the same way to food restriction in development; (2) whether males and females would respond in the same way to food restriction in daily time allocated to foraging and in hunting behavior; and (3) if males and females respond differently to food restriction, when would the difference occur? In order to do these, we compared the development and behavior of males and females under half starvation with a control group and compared the behavioral measurements in both food treatments in juveniles and adults.

## 2 MATERIALS AND METHODS

### 2.1 Study species

**FIGURE 1:**
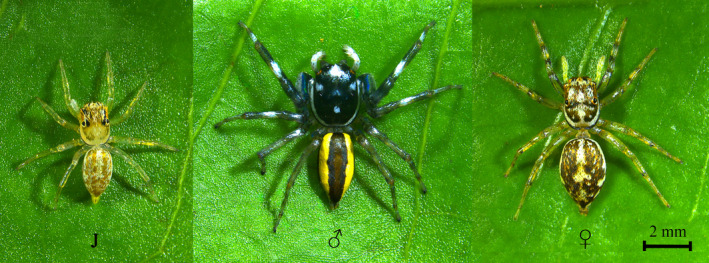
Physical features of *Phintelloides versicolor*. The juvenile, adult male and adult female of *P. versicolor*

*P. versicolor* (Figure 1) were selected for this study for its short developmental period, large population size and wide distribution in tropical Asia. *P. versicolor* are cursorial predators which live in shrubs and feed on small invertebrates. A total of 320 juveniles of *P*. *ver* *sicolor* were collected at Xishuangbanna Tropical Botanical Garden (Yunnan, China) from September to November 2019. Spiders were collected four times on September 20, October 9, October 21 and November 14. Small juveniles (body length between 2 and 3 mm) were collected from the field. Each spider was kept separately in a plastic box (5.4 × 5.4 × 4.2 cm), with a temperature of 25 ± 2℃ with a 12:12 hr light: dark cycle (light: 8:00 – 20:00) and fed with fruit flies (*Drosophila melanogaster*). The food of fruit flies was prepared with the ratio of 200 g corn powder, 200 g sugar, 5 g agar powder, 30 g yeast powder, 1 g benzoic acid and 1 drop of propionic acid. The collected juveniles were randomly assigned into two groups (*N*_starving_ = 161, *N*_control_ = 159): the control group was supplied with abundant food across the entire experiment, and the starving group was provided with only half amount of required food for the entire development process. The specific amount of food spiders required for each life stage were based on pre‐experimental investigations. Accordingly, the numbers of fruit flies required in starving group were one (when spiders were 2–3 mm) and two (when spiders were larger than 3 mm), respectively (Appendix S1 Pre‐experiment).

### 2.2 Experiment 1: Developmental response

In order to investigate the effects of food restriction on the development duration, adult body size and weight and the differences between female and male spiders, we measured the body size of each spider when we started the experiment (“X0_day body length”) and every 5 days since then until they reached adulthood, then their body weight, sex and the development duration. Hatchling *P. versicolor* cannot be reared to adulthood in the lab, and no reports have been found; thus the number of instars is not known, and we estimate there were 5–7 molting before maturation. We measured age by body size, which increases after molting, but we did not record molting. During the experiment, mortality of spiders was recorded every day. In total twenty spiders died before adulthood. Sample size was presented in Table S1.

### 2.3 Experiment 2: Behavioral responses

In order to explore the behavioral responses concerning daily time allocated to foraging (staying inside or outside of the nest) and hunting, of spiders between different food conditions, sexes and growth stages, we randomly selected some spiders (Among the 300 spiders, 213 individuals were randomly selected during the time allocation experiment, and 224 individuals were randomly selected during the predation behavior experiment) from “Experiment 1” and carried out the following trials. Detailed number of spiders from each sex, each growth stage and each food condition are summarized in Appendix S1. (1) To study the time allocation of daily activities, we recorded whether the spider was staying in or out of its nest. *Phintelloides versicolor* uses silk to build a nearly non‐transparent chamber, which can be seen on the surface of natural leaves (in the field) or in the corner of the container internal surface. The nest is used for a long time and the spider only goes out for foraging/sexual activities. Whether a spider was inside of their nest was judged by whether the spider's whole body was inside of the chamber or not. If any part(s) of the body was outside of the chamber, we considered it as outside, which means the spider was active, rather than resting in nest. Every spider was checked four times (twice between 9–11 a.m., and twice between 3–5 p.m.) on the third day after feeding. (2) Concerning hunting behavior, randomly chosen juvenile spiders (reared for 2 weeks or longer) and adult spiders were tested. During the test, each spider was put into a plastic tube (diameter × height: 15 × 80 mm) to get accustomed to the arena for 5 min. Then fruit flies were released into the tube and the opening of the tube was plugged with porous cotton (Juvenile spiders were provided with one fruit fly, while adult spiders were provided with two). The hunting behavior was recorded for 2 hr with video camera SONY FDR‐AX60. We measured the time spent to notice the prey, which the spider spent before directing to the prey with its “face” (looks directly at the prey with its principle eyes whose vision angle is very small so that a turning directed to prey can be easily defined) from the time the prey is placed in, the latency from noticing to the start of hunting and the result (successful or not). All tests were conducted on the feeding day, before feeding the spiders to be sure the spiders were hungry and ready to hunt. The control group was supplemented with sufficient prey after the tests, while the starving group was not (The amount of food eaten during the tests was just the normal feeding amount for the starving group). Some of the individuals (Number of repeats: 1.time allocation of daily activities (*N* = 39); 2. hunting behavior (*N* = 62), including noticing (*N* = 62), success rate (*N* = 62) and latency (*N* = 26)) tested as adults were also tested as juveniles (Table S2).

### 2.4 Statistical analysis

#### 2.4.1 Developmental responses

Generalized linear models (GLMs) were built to find the predictive variables that significantly affected the development duration, adult body length, and weight of the spiders. Development durations data were log‐transformed to meet the normality assumptions of parametric tests. Predictive variables that we were aiming to test in our study are food abundance (control vs. starving), sex, and their interaction; other potential variables that might have effects or we could not keep constant are initial body length of spiders (X0_day body length) and collection batches (1, 2, 3, 4) in our initial models to control their effects. The survival rate between the starvation and control groups was compared by the Fisher's exact test.

#### 2.4.2 Behavioral responses

GLMs were built to find the predictive variables that significantly affected the probabilities of spider staying outside or inside of nest, the probabilities of spider noticing the prey immediately when the prey were released to the testing tube, and the probabilities of spider successfully predating a fruit fly in 2 hr. Predictive variables that were tested in our study are food abundance (control vs. starving), sex, growth stage (juvenile vs. adult), and their interactions. For the probabilities of spider staying outside or inside of nest, we constructed generalized linear mixed models (GLMMs) with binomial error distribution by maximum likelihood (Laplace approximation) using ‘glmer’ function in lme4 package (Bates et al., 2015). In these models, besides the three aiming variables, we also included observation occasions (am1, am2, pm1, pm2), and “spider ID” as random effects, and only for random intercepts. For the probabilities of spider noticing the prey immediately when the prey items were released to their raising box, and the probabilities of spider successfully predating a fruit fly in 2 hr, we used GLMs with binomial error distribution.

#### 2.4.3 Model selection procedures

Model selections were carried out for models on developmental and behavioral responses. We first built a full model including all the potential variables and their interaction terms. Then, to avoid model overfitting due to highly correlated variables or collinearity problems, we checked the variance inflation factor (VIF) values of each predictive terms by ‘check. collinearity’ function of performance package (Daniel et al., 2020) and deleted the terms from the highest order of interaction terms one by one according to their VIF values until all of the VIF values of the remaining terms were less than 3 (Rafael & Raquel, 2020). Next we performed automated model selection using the ‘dredge’ function of the MuMln package (Kamil, 2020) to get a list of models ranked by the Akaike information criterion (AIC) for each model (Burnham et al., 2011). We then derived the model that had the lowest AIC value, calculated, and plotted model predictions by ‘ggeffect’ function of ggeffects package (Daniel, 2018). All analyses were performed in R 4.0.4 (R Core Team, 2021).

## 3 RESULTS

### 3.1 Developmental responses

**FIGURE 2:**
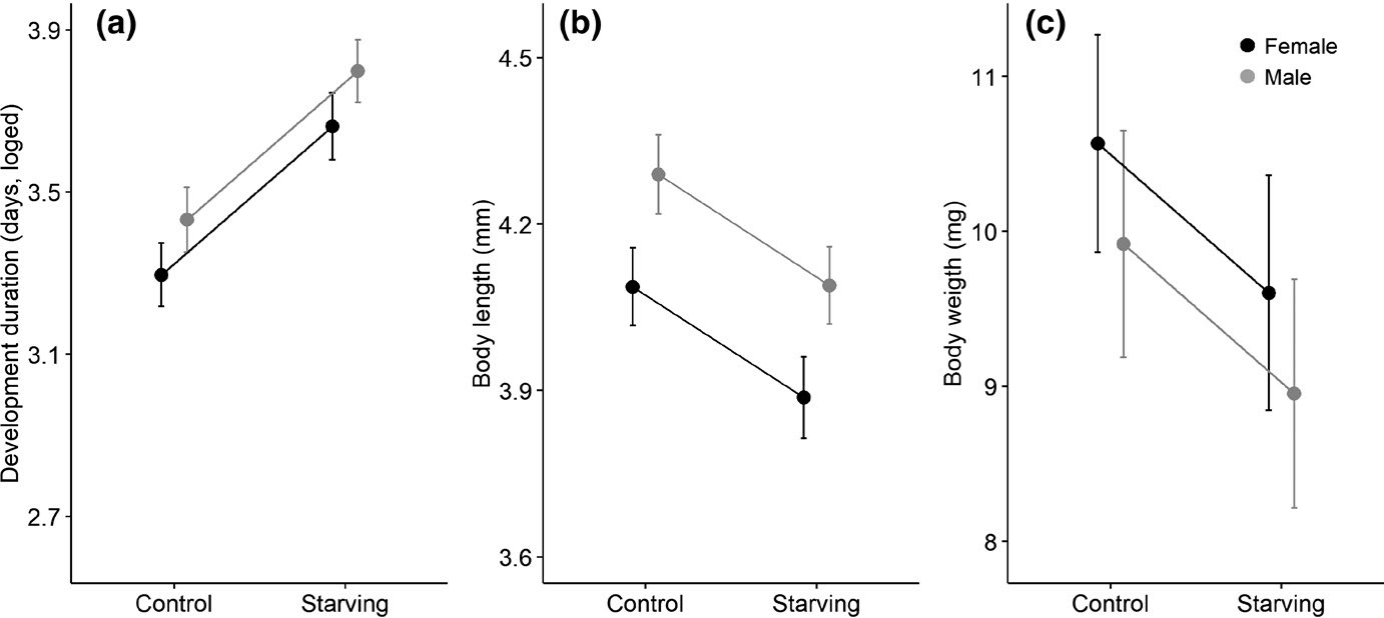
Development duration, adult body length and weight under food restriction and sexual differences. (a) Development duration; (b) Adult body length; (c) Adult body weight

As predicted, starving spiders extended 44.3% of the developmental duration (starving: 41.76 ± 1.03 days, control: 28.95 ± 1.03 days, *t* = 7.90, *p* < .001, Table S3, Figure 2a) and reached adulthood with a 4.8% smaller body size (control: 4.19 ± 0.03 mm, starving: 3.99 ± 0.03 mm, *t* = −4.80, *p* < .001, Table S4, Figure 2b), and a 9.4% lighter body weight (starving: 9.28 ± 0.31 mg, control: 10.24 ± 0.29 mg, *t* = −2.26, *p* = .025, Table S5, Figure 2c) than the control group. However, there was no significant difference in mortality between the two groups (starving: 7%, 14/161; control: 4%, 6/159; *p* = .104, odds ratio = 0.413).

Male and female development responded in the same way to food restrictions; however, there were sexual differences in both development duration and adult body size (not in body mass) (Figure 2, Tables S3, S4, S5). Specifically, males needed 14.6% longer development duration than females (female: 32.31 ± 1.03 days, male: 37.01 ± 1.03 days, *t* = 2.89, *p* = .004, Table S3, Figure 2a). The body size of adult males was 0.2 mm (5.1%) larger than adult females (female: 3.99 ± 0.03 mm, male: 4.19 ± 0.03 mm, *t* = 4.79, *p* < .001, Table S4, Figure 2c), but adult body weight did not differ significantly between sexes (Female: 10.12 ± 0.30 mg, Male: 9.47 ± 0.31 mg, *t* = −1.5, *p* = .135, Table S5, Figure 2c).

### 3.2 Behavioral responses

**FIGURE 3:**
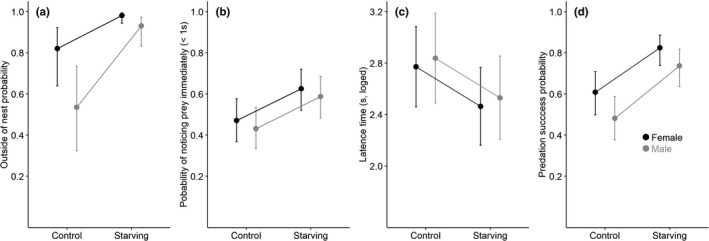
Daily time allocation and hunting behavior under food restriction and sexual differences. (a) The probability of spiders staying out of their nests; (b) The probability of immediately noticing the prey; (c) The latency from noticing to the start of hunting; (d) The probability of successfully subduing the prey

When we examined behavioral responses of the spiders to food restriction in staying outside or inside of their nests and hunting behavior, the responses did not differ between males and females (Figure 3). The starving spiders showed higher probability of staying outside of nest than spiders reared with abundant food (starving: 0.96, 95% CI [0.91, 0.99], control: 0.69, 95% CI [0.51, 0.82], z = 2.47, *p* = .013, Table S6, Figure 3a). The hunting behavior of the spiders under starvation showed a significantly higher probability of noticing the prey immediately (starving: 0.61, 95% CI [0.52, 0.69], control: 0.45, 95% CI [0.37, 0.54], *z* = −2.50, *p* = .012, Table S7, Figure 3b) and about 25.5% shorter latency before hunting (starving: 10.23 ± 1.11 s, control: 13.74 ± 1.14 s, *t* = −2.42, *p* = .017, Table S8, Figure 3c). As predicted, the starving spiders achieved a higher predation success than control individuals (starving: 0.78, 95% CI [0.70, 0.84], control: 0.54, 95% CI [0.46, 0.63], *z* = 4.02, *p* < .001, Table S9, Figure 3d). Therefore, food restriction increased the time spent outside of nest and increased the chances of noticing the prey immediately, reduced latency and increased hunting efficiency.

Although food restriction influenced males and females equally in daily time allocation and hunting behavior, there were sexual differences within food treatment. For daily time allocation, the females had a higher probability to be found staying outside of their nests than males (female: 0.94, 95% CI [0.87, 0.98], male: 0.80, 95% CI [0.66, 0.90], *z* = −3.72, *p* < .001, Table S6, Figure 3a). Although the two sexes did not show significant difference in the probability of noticing the prey immediately (female: 0.55, 95% CI [0.46, 0.63], male: 0.51, 95% CI [0.42, 0.59], *z* = 0.64, *p* = 0. 520, Table S7, Figure 3b) or in latency (“Sex” was excluded from the final model by stepwise model selection, Table S8, while we created Figure 3c based on the model with “Sex” included just to show that there is no effect of “Sex”), the females tended to show a higher hunting success than males (female: 0.73, 95% CI [0.64, 0.80], male: 0.62, 95% CI [0.53, 0.70], *z* = −1.91, *p* = .057, Table S9, Figure 3d).

### 3.3 Age difference on daily time allocation and hunting

**FIGURE 4:**
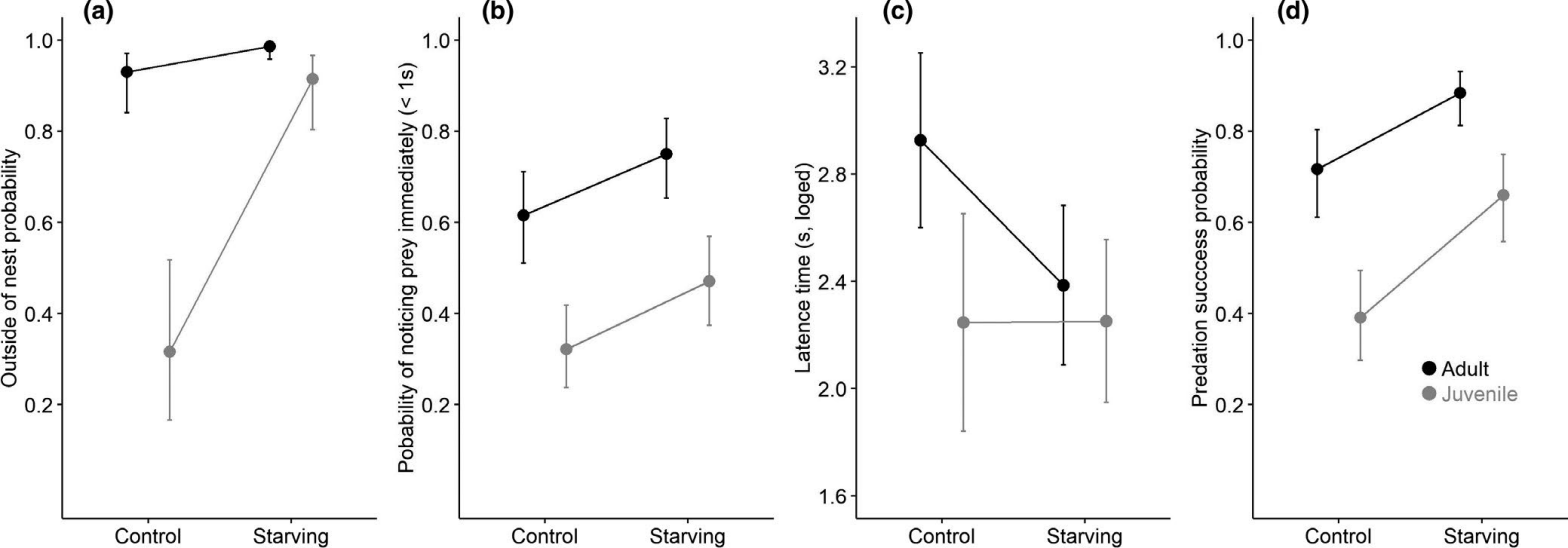
Age difference in daily time allocation and hunting. (a) The probability of spiders staying out of their nests; (b) The probability of immediately noticing the prey; (c) The latency from noticing to the start of hunting; (d) The probability of successfully subduing the prey

Adult spiders had a higher probability to be found outside of their nests than juveniles (adults: 0.97, 95% CI [0.93, 0.99], juveniles: 0.70, 95% CI [0.55, 0.82], *z* = −7.31, *p* < .001, Table S6, Figure 4a). This difference was much stronger when spiders were reared under enough food (adults (control): 0.92, 95% CI [0.84, 0.97], juveniles (control): 0.32, 95% CI [0.17, 0.52]) than those were starving (adults (starving: 0.99, 95% CI [0.96, 1.00], juveniles (starving): 0.91, 95% CI [0.80, 0.97]), which means that there was an interactive effect between food and age (*z* = 2.27, *p* = .023, Table S6, Figure 4a). Furthermore, the interaction between sex and age was also significant (*z* = 3.09, *p* = .002, Table S6), i.e. the difference between adult females (0.99, 95% CI [0.97, 1.00]) and juvenile females (0.75, 95% CI [0.53, 0.88]) were more obvious than between adult males (0.91, 95% CI [0.80, 0.96]) and juvenile males (0.66, 95% CI [0.46, 0.81]).

For hunting behavior, the adult spiders also were more likely to immediately notice the prey items than juveniles (adults: 0.69, 95% CI [0.60, 0.76], juveniles: 0.39, 95% CI [0.32, 0.47], *z* = 4.80, *p* < .001, Table S7, Figure 4b), but the adults spent 43.0% longer time in latency (adult = 13.57 ± 1.12 s, juvenile: 9.48 ± 1.13 s, *z* = −2.57, *p* = .011, Table S8, Figure 4c). Adults achieved a significantly higher predation success rate than juveniles (adults: 0.81, 95% CI [0.74, 0.87], juveniles: 0.53, 95% CI [0.44, 0.61], *z* = −4.83, *p* < .001, Table S9, Figure 4d). Therefore, compared with juveniles, the longer time spent by the adults in latency turned into a higher accuracy, thus increasing hunting efficiency.

## 4 DISCUSSION

We found food restriction equally influenced the developmental and behavioral traits of females and males in *P. versicolor* in our study. The similar degree of plasticity towards food resources indicates the body size of males and females may be under similar extent of selection for condition dependence or canalization. These suggest, although the body size of males and females are believed to be subject to different direction of selection, these selection can result in such a balanced situation, in which SSD is weak and sex‐specific plasticity is absent.

In *P. versicolor*, males had longer development time and longer adult body length, which is not the common cases in spiders (Blanckenhorn et al., 2007). The fact of weak SSD (males were 5% larger while males and females have same weight) in this species showed the importance of body size to males, indicating longer males might be preferred by females or more likely to win a male–male competition (Blanckenhorn, 2005). As female‐biased SSD is the common situation in spiders, there may be relatively stronger sexual selection on male body size than the sexual (if any) and fecundity selection on female body size in *P. versicolor* (Castillo & Núñez‐Farfán, 2008). However, males and females had same body weight; this suggests body shape was selected differently for the two sexes, with male slimmer while females plumper, indicating a selective pressure on fecundity for females (Girard et al., 2021). For daily time allocation and hunting behavior, specifically, females were more likely to stay outside of nests and had higher hunting efficiency than males. The difference in the probability of staying outside of nests between juveniles and adults was stronger in females than that in males. These all suggest that the food requirement of females might be higher than that of males. As reproductive role differs between the sexes, in which males invest energy mostly on mate searching whereas females often in egg production and parental care (Chen et al., 2018; Trivers, 1972), possibly this was due to a higher energy expense during reproduction, which is in line with the finding of larger energy need by females than males in spiders with female‐biased SSD (Walker & Rypstra, 2002). As both males and females *P. versicolor* need to forage actively rather than sit and wait, natural selection may favor females with relatively large body size but also high mobility, which might constrain the evolution of a large body size (Blanckenhorn, 2000), and that may explain why female jumping spiders mostly are not that much larger than males; males may suffer from a reducing cognitive ability if being too small. These suggest though the body size of males and females is favored by different selection forces and some of which are contradictory to each other (body enlargement: sexual selection, fecundity selection; body dwarfing: selection for mobility), the result of these selection can be a weak SSD and same degree of reaction norms of the sexes towards food condition. For the ultimate causes of weak SSD and same degree of reaction norms of the sexes, there may be several explanations. On one hand, these could mean the body sizes could be equally strongly related to fitness for both sexes (equally canalized), the developmental plasticity of which is equally decreased by stabilizing selection or directional selection counteracted by a constraint (Stillwell et al., 2009). On the other hand, this could mean the plasticity of body sizes of both sexes is equally increased so that they showed similar degree of condition dependence (Bonduriansky, 2007), which is selected by directional selection for resource use efficiency, or the lack of sex‐specific responses to food restriction is due to the similar body size between the sexes and the energetic costs during their development are similar. However, as our study did not test which selection each sex are experiencing, studies both testing the plasticity of traits and their effects on fitness and the comparison between the plasticity of multiple traits under different condition (Rohner et al., 2018) are needed to demonstrate which explanation is the case.

Furthermore, we found food restriction induced different responses between life stages in daily time allocation, but not in hunting behavior. Though the probability of staying outside of nests was always higher for adults than juveniles, food restriction increased that probability of juveniles more strongly. These indicate the overall food requirement of adults was higher than that of juveniles, while the stress of food restriction might be higher for juveniles than adults. This may reflect that when food is scarce the trade‐off between food and safety is more biased to food for juveniles than for adults, suggesting the need of development is more urgent than reproduction, or the predation risk is higher for juveniles in the field (Blanckenhorn et al., 2007). Larger size of adults compared with juveniles may reduce the possibility of being hunted by other predators with similar adult body sizes (Nylin & Gotthard, 1998). While for the effects of food restriction on hunting behavior, the equal responses of juveniles and adults suggest the improving potential in hunting efficiency, latency or the probability of noticing the prey immediately is the same for the two age groups. Alternatively, this may also indicate the hunting skills of *P. versicolor* were already developed in the juvenile stage and the difference found in hunting efficiency between juveniles and adults might result from the larger body size rather than the skills.

In terms of the overall effects of food restriction, food‐restricted *P. versicolor* had lower both adult body length and weight than control. However, the plasticity was found in development duration, which might work as a compensatory growth strategy to buffer the decreasing effect of food restriction on body size. In addition, *P*. *versicolor* increased the time spent outside of nest and hunting efficiency when food was restricted. These responses might support a potential higher energy intake if the lacking food resources become more available later. Thus, in addition to extending the development duration, spiders in the field may also incorporate these behavioral adjustments to accelerate growth rate.

In conclusion, we showed both developmental and behavioral responses did not differ between males and females under food restriction in a sexually dimorphic (apparent in color, but weak in size) species, *P. versicolor*. However, when the food was abundant, the sexual differences existed in development, daily time allocation and hunting behavior. These findings suggested, although males and females show significant differences in many life history traits, their plasticity in body size and resource intake strategy can still be selected in a similar degree in sexually dimorphic species with weak SSD.

## CONFLICT OF INTEREST

None declared.

## AUTHOR CONTRIBUTIONS

**Qin Li:** Conceptualization (equal); data curation (lead); formal analysis (lead); investigation (lead); methodology (lead); resources (equal); software (equal); supervision (equal); writing–original draft (lead); writing–review and editing (lead). **Bing Dong:** Data curation (equal); formal analysis (equal); resources (equal); writing–original draft (equal). **Jing‐Xin Liu:** Data curation (equal); formal analysis (equal); resources (equal); software (equal); supervision (equal); writing–review and editing (equal). **Rong Xiao:** Conceptualization (equal); data curation (equal); formal analysis (equal); funding acquisition (equal); investigation (equal); methodology (equal); project administration (equal); resources (equal); software (equal); supervision (equal); validation (equal); visualization (equal); writing–original draft (equal); writing–review and editing (equal). **Zhanqi Chen:** Conceptualization (equal); data curation (equal); formal analysis (equal); funding acquisition (equal); investigation (equal); methodology (equal); project administration (equal); supervision (equal); validation (equal); visualization (equal); writing–original draft (equal).

## Supporting information

Table S1‐S9

## Data Availability

All raw data (about spider development, in nest or out nest observation and predation) used in analyses are available on DRYAD (https://doi.org/10.5061/dryad.8931zcrr0).

## References

[ece38112-bib-0001] Abrams, P. A. (1991). Life history and the relationship between food availability and foraging effort. Ecology, 72(4), 1242–1252. 10.2307/1941098

[ece38112-bib-0002] Aguilar‐Argüello, S. O. , & García‐Chávez, J. H. (2015). Importance of hunger and prey type on predatory behavior stages in *Corythalia albicincta* (Araneae: Salticidae). Journal of Arachnology, 43(2), 143–151. 10.1636/j13-56

[ece38112-bib-0015] Bates, D. , Mächler, M. , Bolker, B. , & Walker, S. (2015). Fitting linear mixed‐effects models using lme4. Journal of Statistical Software, 67(1), 1–48. 10.18637/jss.v067.i01

[ece38112-bib-0003] Berrigan, D. , & Charnov, E. L. (1994). Reaction norms for age and size at maturity in response to temperature: A puzzle for life historians. Oikos, 70(3), 474–478. 10.2307/3545787

[ece38112-bib-0004] Blanckenhorn, W. U. (2000). The evolution of body size: What keeps organisms small? Quarterly Review Biology, 75(4), 385–407. 10.1086/393620 11125698

[ece38112-bib-0005] Blanckenhorn, W. U. (2005). Behavioral causes and consequences of sexual size dimorphism. Ethology, 111(11), 977–1016. 10.1111/j.1439-0310.2005.01147.x

[ece38112-bib-0006] Blanckenhorn, W. U. , Dixon, A. F. G. , Fairbairn, D. J. , Foellmer, M. W. , Gibert, P. , Linde, K. , Meier, R. , Nylin, S. , Pitnick, S. , Schoff, C. , Signorelli, M. , Teder, T. , & Wiklund, C. (2007). Proximate dauses of densch’s dule: Does dexual dize dimorphism in drthropods desult from dex differences in development dime? The American Naturalist, 169(2), 245–257. 10.1086/510597 17211807

[ece38112-bib-0007] Bonduriansky, R. (2007a). The evolution of condition‐dependent sexual dimorphism. The American Naturalist, 169(1), 9–19. 10.1086/510214 17206580

[ece38112-bib-0008] Bonduriansky, R. (2007b). Sexual selection and allometry: A critical reappraisal of the evidence and ideas. Evolution: International Journal of Organic Evolution, 61(4), 838–849. 10.1111/j.1558-5646.2007.00081.x 17439616

[ece38112-bib-0009] Burnham, K. P. , Anderson, D. R. , & Huyvaert, K. P. (2011). AIC model selection and multimodel inference in behavioral ecology: Some background, observations, and comparisons. Behavioral Ecology and Sociobiology, 65, 23–35. 10.1007/s00265-010-1029-6

[ece38112-bib-0010] Castillo, R. C. , & Núñez‐Farfán, J. (2008). The evolution of sexual size dimorphism: The interplay between natural and sexual selection. Journal of Orthoptera Research, 17(2), 197–200. 10.1665/1082-6467-17.2.197

[ece38112-bib-0011] Chen, Z. Q. , Corlett, R. T. , Jiao, X. G. , Liu, S. J. , Charles‐Dominique, T. , Zhang, S. C. , Li, H. , Lai, R. , Long, C. B. , & Quan, R. C. (2018). Prolonged milk provisioning in a jumping spider. Science, 362(6418), 1052–1055. 10.1126/science.aat3692 30498127

[ece38112-bib-0012] Cordellier, M. , Schneider, J. M. , Uhl, G. , & Posnien, N. (2020). Sex differences in spiders: From phenotype to genomics. Development Genes and Evolution, 230(2), 155–172. 10.1007/s00427-020-00657-6 32052129 PMC7127994

[ece38112-bib-0013] Daniel, L. (2018). Ggeffects: Tidy data frames of marginal effects from regression models. Journal of the American Chemical Society, 3, 72. 10.21105/joss.00772

[ece38112-bib-0014] Daniel, L. , Dominique, M. , Philip, W. , Indrajeet, P. , Mattan, S.‐S. , & Philip, W. (2020). Assessment of regression models performance. Comprehensive R Archive. Network. Retrieved from https://easystats.github.io/performance/

[ece38112-bib-0016] Fairbairn, D. J. (1997). Allometry for sexual size dimorphism: Pattern and process in the coevolution of body size in males and females. Annual Review of Ecology and Systematics, 28(1), 659–687. 10.1146/annurev.ecolsys.28.1.659

[ece38112-bib-0017] Fernández‐Montraveta, C. , & Moya‐Laraño, J. (2007). Sex‐specific plasticity of growth and maturation size in a spider: Implications for sexual size dimorphism. Journal of Evolutionary Biology, 20(5), 1689–1699. 10.1111/j.1420-9101.2007.01399.x 17714286

[ece38112-bib-0018] Fischer, K. , & Fiedler, K. (2000). Sex‐related differences in reaction norms in the butterfly *Lycaena tityrus* (Lepidoptera: Lycaenidae). Oikos, 90(2), 372–380. 10.1034/j.1600-0706.2000.900218.x

[ece38112-bib-0019] George, W. U. , Randi, P. , & Beril, K. (2002). Influence of feeding regime on body size, body condition and a male secondary sexual character in *schizocosa ocreata* wolf spiders (araneae, lycosidae): Condition‐dependence in a visual signaling trait. The Journal of Arachnology, 30(3), 461–469. 10.1636/0161-8202(2002)030[0461:IOFROB]2.0.CO;2

[ece38112-bib-0020] Girard, A. , Bréheret, N. , Bal, G. , Mavoungou, J.‐G. , Tchibinda, J.‐F. , Makaya, F. , & Girondot, M. (2021). Unusual sexual dimorphism and small adult size for olive ridley sea turtles are linked to volumetric geometric constraints. Marine Biology, 168(1), 7. 10.1007/s00227-020-03814-7

[ece38112-bib-0021] Hochkirch, A. , & Gröning, J. (2008). Sexual size dimorphism in Orthoptera (sens. str.): A review. Journal of Orthoptera Research, 17(2), 189–196. Retrieved from http://www.jstor.org/stable/25473440

[ece38112-bib-0022] Jackson, R. R. (1977). Courtship versatility in the jumping spider, *Phidippus johnsoni* (Araneae: Salticidae). Animal Behaviour, 25, 953–957. 10.1016/0003-3472(77)90046-X

[ece38112-bib-0023] Kamil, B. (2020). MuMIn: Multi‐Model Inference. R package version, 1.43.17. Retrieved from https://CRAN.R‐project.org/package=MuMIn

[ece38112-bib-0024] Kanesharatnam, N. , & Benjamin, S. P. (2019). Multilocus genetic and morphological phylogenetic analysis reveals a radiation of shiny South Asian jumping spiders (Araneae, Salticidae). ZooKeys, 839, 1–81. 10.3897/zookeys.839.28312 31065224 PMC6482596

[ece38112-bib-0025] Koch, C. L. (1846). Die Arachniden (Vol. Dreizehnter Band). Nürnberg: J. L. Lotzbeck.

[ece38112-bib-0026] Kuntner, M. , & Coddington, J. A. (2020). Sexual size dimorphism: Evolution and perils of extreme phenotypes in spiders. Annual Review of Entomology, 65(1), 57–80. 10.1146/annurev-ento-011019-025032 31573828

[ece38112-bib-0027] Leimar, O. , Karlsson, B. , & Wiklund, C. (1994). Unpredictable food and sexual size dimorphism in insects. Proceedings of the Royal Society of London. Series B: Biological Sciences, 258(1352), 121–125. 10.1098/rspb.1994.0151 7838852

[ece38112-bib-0028] Lenski, R. E. (1984). Food limitation and competition: A field experiment with two carabus species. Journal of Animal Ecology, 53(1), 203–216. 10.2307/4352

[ece38112-bib-0029] Li, J. , Zhang, Z. , Liu, F. , Liu, Q. , Gan, W. , Chen, J. , Lim, M. L. M. , & Li, D. (2008). Uvb‐based mate‐choice cues used by females of the jumping spider *Phintella vittata* . Current Biology, 18(9), 699–703. 10.1016/j.cub.2008.04.020 18450445

[ece38112-bib-0030] Livingston, J. D. , Kahn, A. T. , & Jennions, M. D. (2014). Sex differences in compensatory and catch‐up growth in the mosquitofish *Gambusia holbrooki* . Evolutionary Ecology, 28(4), 687–706. 10.1007/s10682-014-9691-1

[ece38112-bib-0031] Metcalfe, N. B. , & Monaghan, P. (2001). Compensation for a bad start: Grow now, pay later? Trends in Ecology & Evolution, 16(5), 254–260. 10.1016/S0169-5347(01)02124-3 11301155

[ece38112-bib-0032] Morin, J. P. , Moreteau, B. , Pétavy, G. , & David, J. R. (1999). Divergence of reaction norms of size characters between tropical and temperate populations of *Drosophila melanogaster* and *D. simulans* . Journal of Evolutionary Biology, 12, 329–339. 10.1046/j.1420-9101.1999.00038.x

[ece38112-bib-0033] Neumann, R. , Ruppel, N. , & Schneider, J. M. (2017). Fitness implications of sex‐specific catch‐up growth in *Nephila senegalensis*, a spider with extreme reversed SSD. PeerJ, 5, e4050. 10.7717/peerj.4050 29158981 PMC5694211

[ece38112-bib-0034] Nylin, S. , & Gotthard, K. (1998). Plasticity in life‐history traits. Annual Review of Entomology, 43(1), 63–83. 10.1146/annurev.ento.43.1.63 9444750

[ece38112-bib-0051] Oudin, M. J. , Bonduriansky, R. , & Rundle, H. D. (2015). Experimental evidence of condition‐dependent sexual dimorphism in the weakly dimorphic antler fly *Protopiophila litigata* (Diptera: Piophilidae). Biological Journal of the Linnean Society, 116(1), 211–220. 10.1111/bij.12549

[ece38112-bib-0035] Quiñones‐Lebrón, S. G. , Kuntner, M. , & Kralj‐Fišer, S. (2021). The effect of genetics, diet, and social environment on adult male size in a sexually dimorphic spider. Evolutionary Ecology, 35, 217–234. 10.1007/s10682-020-10097-3

[ece38112-bib-0036] R Core Team (2021). R: A language and environment for statistical computing. R Foundation for Statistical Computing. Retrieved from https://www.R‐project.org/

[ece38112-bib-0037] Rafael, R. M. , & Raquel, L. C. (2020). A novel trophobiotic interaction between a Neotropical stink bug and an ant species: Insights into potential benefits to the host plant. Behavioural Processes, 182, 104296. 10.1016/j.beproc.2020.104296 33338575

[ece38112-bib-0038] Rohner, P. T. , Teder, T. , Esperk, T. , Lüpold, S. , & Blanckenhorn, W. U. (2018). The evolution of male‐biased sexual size dimorphism is associated with increased body size plasticity in males. Functional Ecology, 32(2), 581–591. 10.1111/1365-2435.13004

[ece38112-bib-0039] Shine, R. (1989). Ecological causes for the evolution of sexual dimorphism: A review of the evidence. The Quarterly Review of Biology, 64(4), 419–461. 10.1086/416458 2697022

[ece38112-bib-0040] Stillwell, R. C. , Blanckenhorn, W. U. , Teder, T. , Davidowitz, G. , & Fox, C. W. (2009). Sex differences in phenotypic plasticity affect variation in sexual size dimorphism in insects: From physiology to evolution. Annual Review of Entomology, 55(1), 227–245. 10.1146/annurev-ento-112408-085500 PMC4760685 19728836

[ece38112-bib-0041] Stillwell, R. C. , & Fox, C. W. (2007). Environmental effects on sexual size dimorphism of a seed‐feeding beetle. Oecologia, 153(2), 273–280. 10.1007/s00442-007-0724-0 17440751

[ece38112-bib-0042] Teder, T. , & Tammaru, T. (2005). Sexual size dimorphism within species increases with body size in insects. Oikos, 108(2), 321–334. 10.1111/j.0030-1299.2005.13609.x

[ece38112-bib-0043] Tedore, C. , & Johnsen, S. (2012). Weaponry, color, and contest success in the jumping spider *Lyssomanes viridis* . Behavioural Processes, 89(3), 203–211. 10.1016/j.beproc.2011.10.017 22093800

[ece38112-bib-0044] Trivers, R. (1972). Parental investment and sexual selection. Aldine Publishing Company.

[ece38112-bib-0045] Turnbull, A. L. (1965). Effects of prey abundance on the development of the spider *Agelenopsis potteri* (Blackwall) (Araneae: Agelenidae). The Canadian Entomologist, 97(2), 141–147. 10.4039/Ent97141-2

[ece38112-bib-0046] Uhl, G. , Schmitt, S. , Schäfer, M. A. , & Blanckenhorn, W. (2004). Food and sex‐specific growth strategies in a spider. 10.5167/uzh-172731

[ece38112-bib-0047] Vertainen, L. , Alatalo, R. V. , Mappes, J. , & Parri, S. (2000). Sexual differences in growth strategies of the wolf spider *Hygrolycosa rubrofasciata* . Evolutionary Ecology, 14(7), 595–610. 10.1023/A:1011080706931

[ece38112-bib-0048] Vollrath, F. , & Parker, G. A. (1992). Sexual dimorphism and distorted sex ratios in spiders. Nature, 360(6400), 156–159. 10.1038/360156a0

[ece38112-bib-0049] Walker, S. E. , & Rypstra, A. L. (2002). Sexual dimorphism in trophic morphology and feeding behavior of wolf spiders (Araneae: Lycosidae) as a result of differences in reproductive roles. Canadian Journal of Zoology, 80(4), 679–688. 10.1139/z02-037

[ece38112-bib-0050] Weterings, M. J. A. , Moonen, S. , Prins, H. H. T. , van Wieren, S. E. , & van Langevelde, F. (2018). Food quality and quantity are more important in explaining foraging of an intermediate‐sized mammalian herbivore than predation risk or competition. Ecology and Evolution, 8(16), 8419–8432. 10.1002/ece3.4372 30250712 PMC6144975

