# Supplementary material for: Two sexes respond equally to food restriction in a sexually dimorphic but not body mass dimorphic jumping spider
Source: Ecol Evol. 2021 Sep 21;11(20):14033–41. doi: 10.1002/ece3.8112 (PMC8525090; doi:10.1002/ece3.8112)
Supplement: Supplementary file 1 — Table S1‐S9 [file ECE3-11-14033-s001.docx]

**Appendix**

**Supporting file**

1. ***Pre-experiment***

Spiders of different body lengths (such as 2-3mm, 3-4m, 4-5mm, adult female and adult male) were collected from the field. Abundant fruit flies were provided every 5 days, and the maximum feeding amount for spiders was recorded as the food amount required for them to reach satiety. Accordingly, we cut the amount of food supply for satiety by half for food restricting group. According to the preliminary experiment, the following results were obtained: spiders with a body length between 2-3 mm were fed two fruit flies, while others were all supplied with four fruit flies every time. Accordingly, the numbers of fruit flies required in food restriction group were one (2-3 mm) and two (larger than 2-3 mm), respectively.

***2. Sample size in each experiment***

Table S1. Sample size in Experiment 1 developmental responses to food restriction

|  |  | Development duration | Adult body length | Adult body weight | Died Juveniles |
| --- | --- | --- | --- | --- | --- |
| Control  Female  Male  Starving  Female  Male  Total | | 153 | 152 | 124 | 6 |
|  |  | 78 | 78 | 66 |  |
|  |  | 75 | 74 | 58 |  |
|  |  | 147 | 147 | 109 | 14 |
|  |  | 68 | 68 | 52 |  |
|  |  | 79 | 79 | 57 |  |
|  |  | 300 | 299 | 233 | 20 |

Table S2. Sample size in Experiment 2 behavioral responses to food restriction

|  |  | In nest/out nest observation | Noticing fruit fly immediately yes or not | Latency time | Predated a fruit fly or not |
| --- | --- | --- | --- | --- | --- |
| Control  Female  Adult  Juvenile  Male  Adult  Juvenile  Starving  Female  Adult  Juvenile  Male  Adult  Juvenile | | 122 | 145 | 74 | 145 |
|  |  | 59 | 70 | 42 | 70 |
|  |  | 29 | 36 | 28 | 36 |
|  |  | 30 | 34 | 14 | 34 |
|  |  | 63 | 75 | 32 | 75 |
|  |  | 28 | 30 | 17 | 30 |
|  |  | 35 | 45 | 15 | 45 |
|  |  | 130 | 141 | 106 | 141 |
|  |  | 59 | 70 | 54 | 70 |
|  |  | 27 | 30 | 30 | 30 |
|  |  | 32 | 40 | 24 | 40 |
|  |  | 71 | 71 | 52 | 71 |
|  |  | 33 | 33 | 24 | 33 |
|  |  | 38 | 38 | 28 | 38 |
| Total |  | 252 | 286 | 180 | 286 |

***3. Statistical Analysis***

Table S3. Experiment 1: effects of food treatment and sex on development duration

The model with lowest AIC from model selection based on linear models (LM) showing the effect of initial spider body length (X0_day body length), raising food abundance (control versus starving), sex (female versus male) and collection batch (1, 2, 3, 4) on development duration until adult (log transformed to meet the normal requirement of the model).

| Predictors | Estimate | 95% CI | t | *p* |
| --- | --- | --- | --- | --- |
| (Intercept) | 5.16 | 4.80 – 5.52 | 28.06 | <0.001 |
| X0_day body length | -0.70 | -0.80 – -0.59 | -13.46 | <0.001 |
| Food [starving] | 0.37 | 0.28 – 0.46 | 7.90 | <0.001 |
| Collection batch [2] | 0.19 | 0.04 – 0.33 | 2.53 | 0.012 |
| Collection batch [3] | 0.21 | 0.07 – 0.35 | 2.89 | 0.004 |
| Collection batch [4] | 0.38 | 0.18 – 0.58 | 3.75 | <0.001 |
| Sex [male] | 0.14 | 0.04 – 0.23 | 2.89 | 0.004 |
| Observations | 300 |  |  |  |
| R^2^ / R^2^ adjusted | 0.667 / 0.660 |  |  |  |

Table S4. Experiment 1: effects of food treatment and sex on adult body length

The model with lowest AIC from model selection based on linear models (LM) showing the effect of initial spider body length (X0_day body length), raising food abundance (control versus starving), sex (female versus male) and collection batch (1, 2, 3, 4) on adult spider body length (mm).

| Predictors | Estimate | 95% CI | t | *p* |
| --- | --- | --- | --- | --- |
| (Intercept) | 3.8 | 3.47 – 4.12 | 23.05 | <0.001 |
| X0_day body length | 0.12 | 0.03 – 0.21 | 2.55 | 0.011 |
| Food [starving] | -0.20 | -0.28 – -0.12 | -4.80 | <0.001 |
| Sex [male] | 0.20 | 0.12 – 0.29 | 4.79 | <0.001 |
| Collection batch [2] | -0.16 | -0.29 – -0.03 | -2.42 | 0.016 |
| Collection batch [3] | -0.03 | -0.16 – 0.10 | -0.44 | 0.661 |
| Collection batch [4] | -0.13 | -0.31 – 0.05 | -1.44 | 0.150 |
| Observations | 299 |  |  |  |
| R^2^ / R^2^ adjusted | 0.205 / 0.189 |  |  |  |

Table S5. Experiment 1: effects of food treatment and sex on adult body weight

The model with lowest AIC from model selection based on linear models (LM) showing the effect of raising food abundance (control versus starving), growth stage (adult versus juvenile) and sex (female versus male) on adult spider body weight (mg).

| Predictors | Estimate | 95% CI | t | *p* |
| --- | --- | --- | --- | --- |
| (Intercept) | 8.89 | 6.85 – 10.94 | 8.58 | <0.001 |
| X0_day body length | 0.57 | -0.11 – 1.24 | 1.65 | 0.101 |
| Food [starving] | -0.96 | -1.81 – -0.12 | -2.26 | 0.025 |
| Sex [male] | -0.65 | -1.50 – 0.20 | -1.5 | 0.135 |
| Observations | 233 |  |  |  |
| R^2^ / R^2^ adjusted | 0.040 / 0.027 |  |  |  |

Table S6. Experiment 2: effects of food treatment, sex and age on the probability of staying outside of nests

The model with lowest AIC from model selection based on generalized linear mixed effects models (GLMM) showing the effect of raising food abundance (control versus starving), growth stage (adult versus juvenile), sex (female versus male) and observation occasions (am1, am2, pm1 and pm2) on the probability of spider staying outside or inside of nest. A random intercept model was used (1|spiderID was specified as a random factor) to account for the variance among different spiders.

| Predictors | Odds Ratios | 95% CI | z | *p* |
| --- | --- | --- | --- | --- |
| (Intercept) | 80.24 | 22.54 – 285.64 | 6.77 | <0.001 |
| Food [starving] | 5.38 | 1.42 – 20.40 | 2.47 | 0.013 |
| Stage [juvenile] | 0.01 | 0.00 – 0.04 | -7.31 | <0.001 |
| Sex [male] | 0.08 | 0.02 – 0.31 | -3.72 | <0.001 |
| Observation [am2] | 1.01 | 0.56 – 1.83 | 0.02 | 0.980 |
| Observation [pm1] | 0.42 | 0.24 – 0.76 | -2.87 | 0.004 |
| Observation [pm2] | 0.33 | 0.19 – 0.60 | -3.65 | <0.001 |
| Food [starving]:Stage [juvenile] | 4.31 | 1.22 – 15.27 | 2.27 | 0.023 |
| Sex [male]:Stage [juvenile] | 7.65 | 2.10 – 27.85 | 3.09 | 0.002 |
| Random Effects |  |  |  |  |
| σ^2^ | 3.29 |  |  |  |
| τ_00_ _ID_ | 10.06 |  |  |  |
| ICC | 0.75 |  |  |  |
| N _ID_ | 213 |  |  |  |
| Observations | 1008 |  |  |  |
| Marginal R^2^/Conditional R^2^ | 0.243 / 0.813 |  |  |  |

Table S7. Experiment 2: effects of food treatment, sex and age on the probability of noticing fruit fly immediately

The model with lowest AIC from model selection based on generalized linear models (GLM) showing the effect of raising food abundance (control versus starving), growth stage (adult versus juvenile) and sex (female versus male) on whether a spider immediately noticed the fruit fly or not when we put fruit flies into the box.

| Predictors | Odds Ratios | 95% CI | z | *p* |
| --- | --- | --- | --- | --- |
| (Intercept) | 1.74 | 1.07 – 2.88 | -2.19 | 0.029 |
| Food [starving] | 1.88 | 1.15 – 3.09 | -2.50 | 0.012 |
| Sex [male] | 0.85 | 0.52 – 1.39 | 0.64 | 0.520 |
| Stage [juvenile] | 0.30 | 0.18 – 0.48 | 4.80 | <0.001 |
| Observations | 286 |  |  |  |
| R^2^ Tjur | 0.104 |  |  |  |

Table S8. Experiment 2: effects of food treatment, sex and age on latency time before hunting

The model with lowest AIC from model selection based on linear models (LM) showing the effect of raising food abundance (control versus starving), growth stage (adult versus juvenile) and sex (female versus male) on the time (seconds, log transformed to meet the normal requirement of the model) of spiders’ latency time before chasing.

| Predictors | Estimate | 95% CI | t | *p* |
| --- | --- | --- | --- | --- |
| (Intercept) | 2.93 | 2.60 – 3.25 | 17.72 | <0.001 |
| Food [starving] | -0.54 | -0.98 – -0.10 | -2.42 | 0.017 |
| Stage [juvenile] | -0.68 | -1.20 – -0.16 | -2.57 | 0.011 |
| Food [starving]:Stage [juvenile] | 0.54 | -0.13 – 1.22 | 1.6 | 0.111 |
| Observations | 180 |  |  |  |
| R^2^ / R^2^ adjusted | 0.059 / 0.043 |  |  |  |

Table S9. Experiment 2: effects of food treatment, sex and age on the probability of successfully hunting a fruit fly

The model with lowest AIC from model selection based on generalized linear models (GLM) showing the effect of raising food abundance (control versus starving), growth stage (adult versus juvenile) and sex (female versus male) on whether a spider predated and eat fruit fly or not.

| Predictors | Odds Ratios | 95% CI | z | *p* |
| --- | --- | --- | --- | --- |
| (Intercept) | 3.29 | 1.92 – 5.85 | 4.21 | <0.001 |
| Food [starving] | 3.02 | 1.78 – 5.22 | 4.02 | <0.001 |
| Sex [male] | 0.6 | 0.35 – 1.01 | -1.91 | 0.057 |
| Stage [juvenile] | 0.25 | 0.14 – 0.44 | -4.83 | <0.001 |
| Observations | 286 |  |  |  |
| R^2^ Tjur | 0.15 |  |  |  |
